# Perspectives on the application of nanotechnology in photodynamic therapy for the treatment of melanoma

**DOI:** 10.3402/nano.v5.24381

**Published:** 2014-09-01

**Authors:** Victoria Monge-Fuentes, Luis Alexandre Muehlmann, Ricardo Bentes de Azevedo

**Affiliations:** Laboratory of Nanobiotechnology, Department of Genetics and Morphology, Institute of Biological Sciences, University of Brasília, Brasília-DF, Brazil

**Keywords:** photodynamic therapy, skin cancer, melanoma, nanoparticles, nanotechnology

## Abstract

Malignant melanoma is the most aggressive form of skin cancer and has been traditionally considered difficult to treat. The worldwide incidence of melanoma has been increasing faster than any other type of cancer. Early detection, surgery, and adjuvant therapy enable improved outcomes; nonetheless, the prognosis of metastatic melanoma remains poor. Several therapies have been investigated for the treatment of melanoma; however, current treatment options for patients with metastatic disease are limited and non-curative in the majority of cases. Photodynamic therapy (PDT) has been proposed as a promising minimally invasive therapeutic procedure that employs three essential elements to induce cell death: a photosensitizer, light of a specific wavelength, and molecular oxygen. However, classical PDT has shown some drawbacks that limit its clinical application. In view of this, the use of nanotechnology has been considered since it provides many tools that can be applied to PDT to circumvent these limitations and bring new perspectives for the application of this therapy for different types of diseases. On that ground, this review focuses on the potential use of developing nanotechnologies able to bring significant benefits for anticancer PDT, aiming to reach higher efficacy and safety for patients with malignant melanoma.

The incidence of melanoma has been continuously increasing over the past decades to the extent that it is now reaching epidemic proportions in white populations worldwide (1, 2). According to the latest report by the World Health Organization, approximately 232,130 new cases of melanoma skin cancer occurred globally in 2012 (3). Moreover, the latest American Cancer Society report (2013) states that about 76,690 new melanoma cases were to be diagnosed and about 9,480 people were expected to die of melanoma in 2013 (4).

Malignant melanoma is the most aggressive form of skin cancer and develops as a result of the combination of both genetic and environmental factors (5, 6). This type of cancer is generated by malignant phenotypes of the skin melanocyte, a cell type that originates from the developing neural crest and migrates to the skin, hair follicles, eyes, and ears (6). In the skin, the melanocyte resides in the basal layer of the epidermis surrounded by approximately 36 keratinocytes – a compartment referred to as the epidermal-melanin unit (EMU). In the EMU, the melanocyte produces the pigment melanin via the enzymatic process of melanogenesis in specialized intracellular organelles called melanosomes (7). Presence of this cell-specific organelle – the melanosome – and its associated product, melanin pigment, set melanoma apart from all other types of cancers. Thus, treatments targeting melanoma should strongly consider the melanosome to successfully fight this type of cancer (8).

Melanoma can be classified into three categories: cutaneous melanoma (91.2%), ocular melanoma (5.3%), and mucosal melanoma (1.3%) (9). Ocular melanoma, also known as uveal melanoma or choroidal melanoma, is the most common primary intraocular malignant tumor (10). Mucosal melanoma can occur in any mucous membrane of the body, including the nasal cavity and accessory sinuses, oral cavity, anorectum, and others (11). Cutaneous melanoma is the most common type, known as a heterogeneous disease with many clinicopathologic subtypes. Of these, the majority fits into four categories: superficial spreading melanoma (SSM), nodular melanoma (NM), lentigo maligna (LM), and acral lentiginous melanoma (ALM), comprising 70–75%, 20–25%, 5–10%, and 5% of cutaneous melanoma cases, respectively, in White Caucasian populations (12). However, acral melanomas represents the most prevalent histologic subtype in African, African American, Chinese, Taiwanese, and mixed racial heritage populations (13). Besides histopathological differences among these subtypes of cutaneous melanoma, other clinical factors differentiate them. LM begins as a tan macule that extends peripherally, with gradual uneven darkening over the course of years and tends to be more common in older patients with heavily sun-damaged skin. Unlike LM, SSM has no preference for sun-damaged skin, being associated with intermittent and sporadic sun exposure. The upper back in both sexes and the legs in women are the most common sites. There is a tendency to multicoloration, not just with different shades of tan, but variations of black, red, brown, blue, and white. ALM appears more commonly in the foot, demonstrates a junctional growth pattern, indistinct margins, and over time, a vertical growth phase develops and shows little association with sun exposure. NM presents lesions that arise without a clinically apparent radial growth phase, but usually large atypical melanocytes can be found in the epidermis beyond the region of vertical growth. Tumors appear primarily on sun-exposed areas of the head, neck, and trunk, and may be smooth and dome-shaped, fungating, friable, or ulcerated. Bleeding is usually a late sign (13, 14).

Melanomas are also classified in relation to melanin content. Most types of melanomas are melanotic, containing various degrees and types of pigmentation (melanin); however, any clinical subtype of primary cutaneous melanoma or metastatic melanoma may be amelanotic, presenting the absence of pigmentation in the tumor. Amelanotic melanoma represents 1.8–8.1% of all such tumors (15).

Unfortunately, rapid increase in malignant melanoma incidence has not been paralleled by the development of better therapeutic options over the last decades (16). Patients with early-stage, non-metastatic melanoma can be treated by the surgical removal of the tumor, with high survival rates. For instance, patients with removable melanoma were treated with chemosurgery, reaching a cure rate of 97–99.8% (17, 18). However, the scenery is quite different for metastatic melanoma. Once melanoma has developed into late-stage, metastatic disease, it is difficult to treat, resulting in high mortality (1, 4, 19). Median survival time of patients with unresectable melanoma, depending on the patient's performance status, as well as location and number of metastases, is only about 2–8 months (19). Available treatments, such as chemotherapy, radiotherapy, and immunotherapy, have shown little effect against metastatic melanoma, while causing serious health-threatening effects related to the lack of specificity for tumor cells (20, 21). In the case of patients with unresectable melanoma under conventional treatments, a 3-year overall survival rate of less than 15% is generally observed (1, 4).

Currently, the standard reference drug for chemotherapy in patients with advanced melanoma is dacarbazine (DTIC), the first-line treatment for patients with melanoma (22), showing response rates of 15–25%, with median response durations of 5–6 months, and less than 5% of complete responses (19). These grim outcomes are due to problems frequently stated for dacarbazine, such as its low chemical stability, rapid metabolization following intravenous administration, melanoma resistance, and negative adverse effects (23). Attempts to improve the therapeutic index of dacarbazine with nanotechnological tools have been described in literature (24–26). In these studies, better dacarbazine anticancer activity was obtained when the drug was incorporated into nanoformulations, attributing greater drug stability conferred by nanostructures (24–26) and to alleged size-dependent modification of drug pharmacokinetics (24–26). However, to the authors’ knowledge, so far there are no clinical studies reporting the use of dacarbazine in nanoformulations.

Considering results obtained for melanoma patients treated with dacarbazine, it becomes clear that improved melanoma diagnosis methods and new treatments with higher therapeutic indices are needed. Photodynamic therapy (PDT) has been pointed out over the last years as a candidate for improving melanoma treatment (27), as well as other skin cancer types. At the same time, other studies have additionally investigated as to whether some of the tools available in PDT could also be of use for melanoma diagnosis (28). However, PDT still presents a series of limitations that interfere with its capacity to efficiently treat cancer (29–31).

Nanotechnology has recently been considered as a tool to counter these drawbacks (32–35). In general terms, the application of nanotechnology in PDT aims to improve water compatibility of hydrophobic drugs/photosensitizer (PS), protect the drug from degradation, produce a prolonged release of the drug, increase drug bioavailability (35), increase tumor selectivity, and permit greater penetration depths for the treatment of deep seated tumors, thus increasing treatment efficacy and reducing side effects (36). In view of these factors, the focus of this review is to propose and discuss the potential use of developing nanotechnologies able to bring important benefits for anticancer PDT aiming to reach higher efficacy and safety for melanoma theranostics.

## Photodynamic therapy

**Fig. 1:**
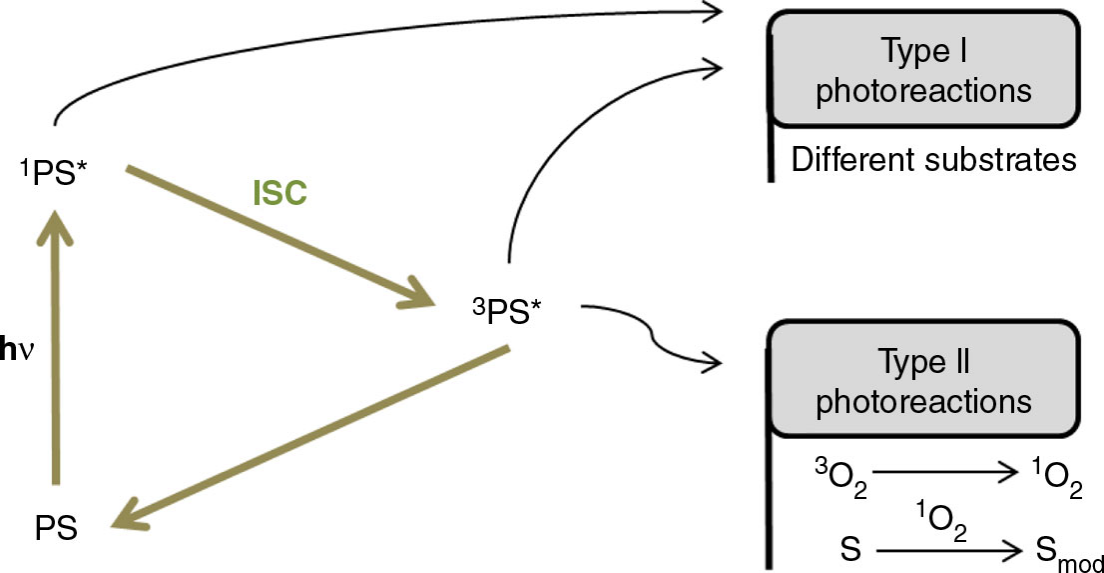
Diagram illustrating the main events leading to photoreactions of type I and II that ultimately may lead to oxidative cell damage. PS=photosensitizer; ^1^PS*=singlet excited state PS; ^3^PS*=triplet excited state PS; hν=photon; ISC=intersystem crossing; ^3^O_2_=triplet oxygen; ^1^O_2_=singlet oxygen; S=substrates (biomolecules); S_mod_=chemically modified biomolecules.

PDT represents a promising minimally invasive therapeutic procedure that employs two individually non-toxic components that are combined to induce a strong oxidative stress in a biological target, intended for the treatment of a variety of solid tumors and non-malignant lesions (37, 38). One of the components is light of a specific wavelength, while the other one is a photosensitizer – a molecule that converts light energy into chemical potential. Once the PS absorbs light, it reaches a singlet excited state and, eventually, it may reach, by intersystem crossing, a relatively stable energetic state called the excited triplet state. PS in its excited triplet state may then directly react with different surrounding molecules – type I photoreactions – or react with molecular oxygen – type II photoreactions. As molecular oxygen is present in tumor sites, type II photoreactions are of particular interest in anticancer PDT. In type II photoreactions, PS promotes, in the presence of molecular oxygen, the transition from the triplet ground state (^3^O_2_) to the singlet excited state (^1^O_2_) (34). These events are summarized in Fig. 1.

In a biological medium, ^1^O_2_ promptly reacts with several surrounding biomolecules, such as proteins, lipids, and nucleic acids (36). This oxidative damage to biomolecules may lead to three biological events that are highly relevant in anticancer PDT: 1) direct cancer cell death by necrosis, apoptosis, or autophagy; 2) tumor ischemia following PDT-induced vascular damage; and 3) activation of the immune system against tumor antigens. Moreover, these three biological mechanisms of PDT may interact with the mechanisms of other chemotherapeutic drugs, raising the possibility of applying PDT along with other drugs in a single anticancer therapy. For example, Castano *et al*. showed that the combination of low-dose cyclophosphamide therapy and PDT, both known to activate and boost the immune response against tumors, led to the elimination of highly metastatic J774 sarcoma cells in a mouse model, which was not observed when these two therapies were applied isolated (39).

As the cytotoxic mechanism of PDT involves PS activation by light of specific wavelength in order to produce oxidative species, the phototoxic effects of PDT and the safety of this procedure may be restricted to the tumor site by simply focusing the light beam on the target area. Thus, PDT represents a site-specific treatment triggered by local light activation (40, 41). In the case of the skin, since it is the tissue most probably exposed to sun light or light emitted by other sources, the issue of skin photosensitization is the most common negative effect observed in PS, as reviewed elsewhere (40). These adverse effects to non-target areas are generally related to exposure of these sites to non-therapeutic light, instead of being related to the toxicity of the PS itself in the absence of light (41). However, this side effect is not so therapy limiting if compared to those elicited by other chemotherapeutic drugs, mainly when the patient is properly advised to avoid exposition to light during the period of treatment. In the case of PS used in clinical practice, such as 5-aminolevulinic acid (ALA), which is applied topically, and methyl aminolevulinate (MAL) applied intravenously (i.e. Phoscan and Photofrin), further specificity to cancer is reached because these molecules are pro-drugs, and thus need to be metabolized into active PS, protoporphyrin IX (PpIX), before light is applied. As cancer cells are generally more active in converting ALA and MAL to PpIX, they concentrate more PpIX in comparison to normal cells (42). Other methods for increasing specificity of PDT to tumors were also described elsewhere (43). Nanotechnology may increase the accumulation of PS in tumor tissue, as described in this review.

Several studies have demonstrated PDT as a viable treatment option against early-stage cancer such as esophageal dysplasia (44), lung cancer (45), head and neck cancer (46–49), non-melanoma skin cancer (50), anal cancer (51), peritoneal ovarian cancer (52), and others. PDT has also been successfully used as co-therapy in late-stage cancer and also has been shown as clinically effective for the treatment of non-cancerous skin lesions, such as recalcitrant warts, acne vulgaris, and psoriasis (53). Still, some obstacles in the clinical adoption of PDT for the treatment of cancer persist (9, 39). In a way to counter these problems, more recently, studies have reported the use of PDT combined with other therapies, such as cryotherapy (54) and carbon dioxide laser (55) for the treatment of nodular basal cell carcinoma skin cancer, demonstrating good efficacy and satisfactory cosmetic outcomes. Nevertheless, when it comes to the use of PDT against melanoma, some particularities of this type of cancer raise important questions that need to be taken into account.

## PDT for the treatment of melanoma

As previously discussed, PS activation by light in the presence of molecular oxygen represents a crucial event in PDT. Ideally, light used in PDT should be poorly absorbed by biological tissues and strongly absorbed by the PS. Biological tissues generally show low absorption of light with wavelengths between 650 and 800 nm and, therefore, this range is called the optical window of biological tissues. In the case of cutaneous melanomas, however, the scenery is different due to the presence of a high content of melanin (56, 57).

**Fig. 2:**
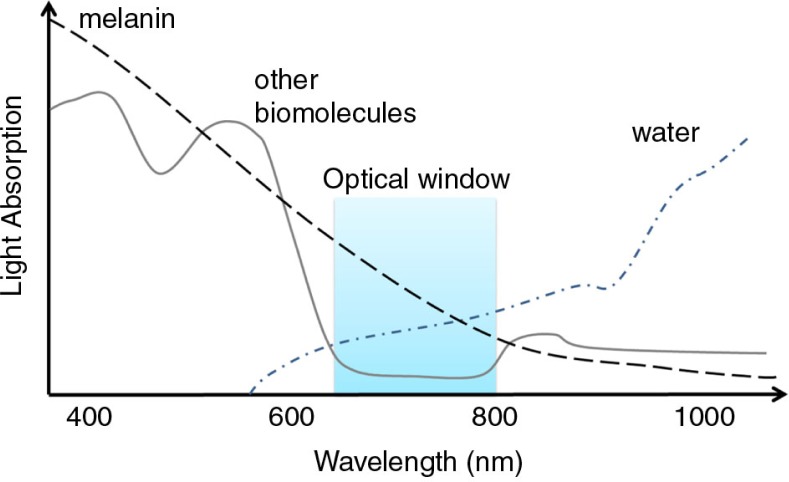
Contribution of different compounds to the optical density of biological tissues.

Melanin is a skin photoprotectant that absorbs light over the entire wavelength region used for PDT (400–750 nm) (57), presenting stronger absorptions in the shorter wavelengths of this spectral range (16) (Fig. 2). Hence, melanin competes with PS for the light used in PDT. Consequently, melanotic melanomas have commonly presented a poor response to classical PDT. In order to prove that melanin is in fact a major obstacle for PDT treatment, several studies have compared experimental results with pigmented (such as B16F1 and B16F10) and depigmented cell lines (for instance, A_375_) (58, 59) or normal melanoma cell lines with photobleached melanin (57). These studies have reinforced that the lack of pigmentation in melanomas decreases resistance to cell death after PDT treatment (60).

A series of modifications and improvements applied on classical PDT have led to better results for melanoma treatment. Studies with PS absorbing in the far-red region suggest that a high level of skin pigmentation is not an obstacle for applying PDT against pigmented tumors, such as B16 pigmented melanoma (61, 62). However, as stated by Bechet and collaborators (32), the majority of existent PS have not yet made it to clinical trials due to factors such as poor selectivity in terms of target tissue and healthy tissue, low extinction coefficients, absorption spectra at relatively short wavelengths, and high accumulation rates in the skin. Most PS that are commercially available present specific beneficial characteristics, but so far none of these incorporate all the properties of an ideal PS, which should present: a stable composition, minimal self-aggregation tendency, non-toxicity in the absence of light exposure, photostability, quick clearance from the body, and target specificity, among other characteristics (32). Thus, as observed for different cancer treatments, the use of nanotechnology as a tool for the treatment of melanoma through PDT becomes a very promising approach, since proper preparation and application of these nanomaterials could satisfy all or at least most of the requirements for an ideal photosensitizing agent.

## Nanotechnology as a tool for improved anticancer efficiency

Nanotechnology is related to the understanding and control of matter at dimensions roughly in the range of 1–100 nm, a scale at which unique chemical and physical properties of materials emerge, allowing to develop, for instance, novel diagnostic and treatment modalities (63–67).

In the case of melanoma, a range of nanoparticle formulations could be envisaged to act as carriers of conventional chemotherapeutic drugs, such as dacarbazine, paclitaxel, docetaxel, doxorubicin, cisplatin, vincristine, and carboplatin (1, 68). More recently, in 2011, the U.S. Food and Drug Administration (FDA) approved Zelboraf (vemurafenib) and Yervoy (ipilimumab) (69), pharmaceuticals that are poised to become the therapy of choice for patients with previously untreated advanced melanoma (70). Moreover, considering that approximately half of melanomas that arise in the skin present a BRAF gene mutation (71), the FDA approved in 2013 the use of two new drugs, Tafinlar (dabrafenib) and Mekinist (trametinib), for patients with unresectable melanoma (72). Initially, in 2013 the FDA approved these drugs to be used as single agents, not as a combination treatment (72). Nonetheless, new information released in January of 2014 declared that the FDA now approves Mekinist (trametinib) in combination with Tafinlar (dabrafenib) to treat patients with advanced melanoma that is unresectable or metastatic (late-stage) (73). Some of these chemotherapeutics have already been nanoencapsulated in liposomes, dendrimers, polymersomes, carbon-based, inorganic, and protein-based nanoparticles (22), which may be used in the future for the treatment of melanoma.

Generally, compared with conventional drug delivery approaches, nanoparticle-mediated delivery of anticancer chemotherapeutic drugs has been reported as a way to improve pharmacokinetic properties of compounds. This is attributable to better tumor accumulation by passive and active targeting, sustained drug release, and prolonged blood circulation times (1).

## Nanotechnology applications for PDT

The use of nanotechnology has provided many tools that can be applied to PDT, bringing new perspectives for the application of this therapy for different types of diseases, such as melanoma. For instance, lipid-based nanoparticles (NPs) (36, 74), polymer-based NPs (75), polyethylene glycol (PEG)-lipid micelles (76), silica-based NPs (60, 77), gold NPs (78–80), upconversion nanoparticles (UCNPs) (81), iron oxide NPs (82–84), nanocapsules (85–87) and fullerenes (88, 89) have already been applied in the treatment of melanoma in *in vitro* and *in vivo* experimental models, showing interesting results (Table 1).

**Table 1:** Types of nanomaterials used with Photodynamic Therapy (PDT) for the treatment of melanoma in in vitro and in vivo experimental models. Benefits and drawbacks related to the application of each type of nanomaterial are also cited.

Nanomaterial	Photosensitizer (PS) incorporated into the nanomaterial	Benefits	Drawbacks
**Lipid-based NPs**			
Liposomes	Liposomes+chloroaluminum phthalocyanine (74)	- Versatility - PS protection - Drug delivery - Most extensively studied type of nanocarrier system - Prevents PS aggregation - Works with hydrophobic and hydrophilic PS (36) - Permits the use of lower concentration of the PS and lower light doses than those applied in current protocols (74)	- Short plasma half-life - Opsonization by plasma proteins and reticulum endothelial system (RES) (36)
**Polymer-based NPs** Poly (lactic-*co*-glycolic acid) (PLGA)	PLGA+meso Tetraphenylporpholactol (m-TPPPL) (75).	- Ability to deliver large amounts of PS to target area - Flexibility toward surface modification for better efficiency - Ability to prevent degradation in the living biological environment - Possibility of being loaded with multiple components such as targeting ligands and contrast agents (36) - Easy to formulate - Biodegradable - Biocompatible - Stable - Non-phototoxic upon systemic administration - Upon cellular internalization, the PS is released from the NP and becomes highly phototoxic (36, 75)	- Tendency to be taken up by macrophages after intravenous administration (36)
**Polyethylene glycol (PEG)-lipid micelles**	Meso-5,10,15,20-tetraphenyl-21H,23H-porphine (TPP) (76).	- PEG prevents rapid uptake of the particles by RES (36) - PEG-PE micelles allowed a 150-fold increase in the solubilization of TPP, compared with the free drug (76)	
**Silica-based NPs (SiNPs)**	Silicon phthalocyanine 4 (Pc4) (60)	- Large surface area and pore volume allow for high drug loading - Tunable diffusional release of drug molecules from the highly ordered mesoporous structure gives rise to a biogenic local concentration at the targeted area, reducing the overall dosage and preventing any acute or chronic complications - Offer the ability to effectively protect the pharmaceutical cargoes from premature release and the undesired degradation in harsh environments (77) - A variety of precursors and methods are available for their syntheses, allowing flexibility and thus numerous PDT drugs can be encapsulated - SiNPs also have advantages as drug vectors; compatibility in biological systems and are not subject to microbial attack (36) - Offer the possibility to functionalize their surface with stimuli-responsive groups for controlled release of various cargos (77) - Encapsulation of Pc4 in silica NP improved the aqueous solubility, stability, and delivery of the photodynamic drug, increased its photodynamic efficacy compared to free Pc4 molecules (60)	- Factors such as surface area and size distribution, chemical composition, surface structure, solubility, shape, and aggregation are potentially toxic triggers (77)
**Gold NPs**	Zn(II)-phthalocyanine disulphide (C11Pc) (78)	- Improved targeting effect - Small size permits enhanced permeation of tumor tissue and vasculature - Chemical inertness - Minimum toxicity - Can serve both as diagnostic and therapeutic tools for cancer - Versatile surfaces - Tunable sizes - Unique optical properties - Can be coated with PEG serving as drug delivery systems for PDT (stabilization by steric repulsion and inhibition of colloid aggregation in physiological conditions) (36, 79) - PDT studies with the C11Pc-loaded amelanotic melanoma showed extensive damage of the blood capillaries and the endothelial cells (78)	- Toxicity in live human subjects; ultimate destination, and possible pathways; and mechanisms for their absorption, circulation, distribution, metabolism, and excretion (79)
Shells of charged biocompatible polymers grafted on gold nanospheres (80).	Dibromobenzene-based chromophore (DBB) (80)		
**Upconversion nanoparticles (UCNs)**	Mesoporous silica–coated upconversion fluorescent nanoparticles (UCNs) loaded with merocyanine (MC540) and zinc (II) phthalocyanine (ZnPc) (81)	- Monodispersibility - Controllable size of less than 100 nm - Noninvasive imaging of deep tissues - Drug delivery - Allows existing photosensitizers to overcome the limited penetration of their activation light and potentially attain their full therapeutic potential (36)	- Unstable attachment and low attachment efficiency of the photosensitizers to the UCNs when shells are not used (36, 81)
**Iron oxide nanoparticles (IONPs)**	- Nanocomposite: IONSPs +fullerenes+PEG+hematoporphyrin monomethyl ether (82) - Iron oxide magnetic core coated with a biocompatible dextran shell bearing polyaminated chlorin p6 (83)	- Strong superparamagnetism and powerful photodynamic therapy capacity (83) - Promising tool for the site-specific delivery of drugs and diagnostic agents by an external magnetic field applied outside the body (36)	- Opsonization; particle aggregation; potential disturbance in iron homeostasis; biodegradability; biocompatibility (84)
**Nanocapsules**	- Nanocapsules containing chloroaluminum phthalocyanine (ClAlPc) (85)	- Nanoencapsulation enables application of hydrophobic photosensitizers with the use of both low sensitizer drug concentration and light dose (85) - Polymeric shell protection against degradation factors like pH and light and the reduction of tissue irritation due to the polymeric shell (86)	- When the drug or PS is entrapped, it has to be added before or during the formulation process, and is thus likely to be degraded (87)
**Fullerenes**	- Fullerene is the actual PSi.e. D-glucose residue pendant fullerene (88)	- Potent agents in photodynamic therapy and magnetic imaging - Excellent triplet sensitizers - Capable of reacting with a wide range of biological targets and killing cancer cells (89)	- Poor in terms of solubility in commonly used organic solvents - Insoluble in aqueous media (89)

According to Paszko and collaborators (34), most current studies on nanotechnology for PDT are aimed at either improving existing formulations of clinically approved PS or are focused on the development of targeted delivery vehicles. Application of nanotechnology in PDT may also aim to improve the solubility of poorly water-soluble drugs, protect the drug from degradation, produce a prolonged release of the drug, and increase drug bioavailability (35, 90). Other advantages presented by nanoparticles include multidrug loading capacity, facilitating combination therapy; and design of NPs to have multiple functions, such as targeting to cancer cells and at the same time permit image contrast (22).

Some of the main possibilities raised by nanotechnology regarding necessary improvements for anticancer PDT to reach higher efficacy and safety are discussed in more details below.

### Delivering the PS to its action site

As for other anticancer drug therapies, PDT efficacy and safety can be improved by increasing the amount of drug reaching the tumor site while decreasing its concentrations in non-target tissues. The first experimental chemotherapeutic drug incorporated in nanocarriers were tested in the 1980s, when initial results already demonstrated that better therapeutic indexes could be reached for classical anticancer drugs by associating them with nanostructured drug delivery systems (91–93). Some of these nanostructured systems are known to accumulate passively in some kinds of solid tumors due to the enhanced permeation and retention (EPR) effect, which is a consequence of defective microvasculature and lymphatic drainage observed in several solid tumors (34, 94). Blood capillary vessels in tumor tissue may have fenestrae of about 200 and 800 nm in diameter, much larger than those observed in the microvasculature of normal tissues (34, 94). Experiments showing the size-dependent accumulation of particles in tumor tissues (95) demonstrate that size matters in anticancer chemotherapy. Thus, by using nanoparticles of an adequate size, it is possible to increase PS concentration in tumor tissue while reducing it in normal tissues (32, 78, 96, 97).

It is also possible to increase the accumulation of drugs in tumors by means of active targeting strategies. Molecules such as antibodies, cationic peptides, agonists of membrane receptors, and others, can be attached to the surface of drug-loaded nanoparticles, increasing their affinity for tumor tissue (98–100). This strategy was already applied in drug delivery systems intended for PDT application. For instance, biocompatible block co-polymer micelles containing Pc4 PS were surface-modified with epidermal growth factor receptor (EGFR)-targeting GE11 peptides for active targeting of EGFR-overexpressing cancer cells. These micelles were incubated with epidermoid carcinoma cells, later treated with PDT. Results indicated that active targeting with EGFR accelerated intracellular PS uptake, enhancing PDT-induced cytotoxicity (97). Moreover, new conjugates consisting of nanobodies (NB) targeting the EGFR and a traceable PS were studied. Results showed that these conjugates specifically induced cell death of EGFR overexpressing cells in low, nanomolar concentrations, while PS alone or NB–PS conjugates in the absence of light induced no toxicity (101).

PDT efficiency was also demonstrated in folic-acid conjugated graphene oxide loaded with chlorin e6 when treating human stomach cancer MGC803 cells (102). Yoon and collaborators (103) also tested chlorin e6 in tumor-targeting hyaluronic acid nanoparticles (HANPs) used as nanocarriers, resulting in effective tumor growth suppression. Nuclei-targeting systems have also been tested through the use of an easily fabricated yet entirely biocompatible and inexpensive polysaccharide-functionalized nanoscale lipid carrier, which triggers the intracellular release of PS inside cancer cells and targets cell nucleus to achieve a significantly enhanced photocytotoxicity. This system may contribute to the development of a new generation of PS carriers that fight against deep-seated tumors and exhibit excellent photodynamic efficiency under faint light irradiation (104).

### Improving the pharmacokinetics of PS

Several PS for anticancer PDT demonstrate a strong hydrophobic nature (34). Such is the case for various PS that have been clinically approved. This hydrophobicity presents positive and negative aspects. The positive point is the higher accumulation and retention of hydrophobic PS in tumor cells (105, 106). This may be due to the fact that hydrophobic PS (i.e. hypericin) associates with serum lipoproteins, such as low density lipoproteins (LDL) (107), which are avidly taken up by tumor cells (108). As discussed by Davids and Kleemann (30), this fact is further supported by a study conducted by Ho and colleagues (109) showing that cholesterol serves as a key determinant for the uptake of hypericin into cellular membranes.

On the other hand, hydrophobic PS readily aggregates in water, hampering the administration of the drug *in vivo*, and also dramatically losing photodynamic activity (29, 31, 90). This may be a major limiting factor for PDT potential clinical application. In this context, it is known that nanomaterials can be used for dispersing hydrophobic PS in biological media. For instance, in a study conducted by Lima and collaborators (110), encapsulation of hypericin in solid lipid nanoparticles improved photodynamic efficiency of this PS, showing a 30% increase in cell uptake and a correlated improvement of 26% in cytotoxicity. In another study, Muehlmann and collaborators (90) showed that the photodynamic activity of aluminum-phthalocyanine chloride (AlPc), a highly effective though extremely hydrophobic phthalocyanine derivative, was significantly improved in aqueous media by associating it with poly(methyl vinyl ether-co-maleic anhydride) nanoparticles. When associated with these polymeric nanoparticles, AlPc had its light-triggered singlet oxygen generation capacity increased by 10-fold in aqueous medium compared to its free form (90).

### Exploring beyond the visible: UCNPs

Some researchers have explored optical properties of nanomaterials in order to circumvent the problem of light absorption by melanin in PDT (99, 111, 112). Particularly interesting, a type of nanomaterial known as upconversion nanoparticles participates in a non-linear optical process that converts two or more low-energy pump photons from the near-infrared (NIR) spectral region (700–1100 nm) to a higher-energy output photon with a shorter wavelength. In simpler terms, UCNPs serve as nanotransducers that convert deeply penetrating NIR light, which does not have enough energy to promote the triplet to singlet state conversion in molecular oxygen, into visible light (VIS) (81). VIS light converted by the UCNPs is then transferred to an appropriate PS with an excitation band matching the emission of the UCNPs. Upon excitation of UCNPs with NIR light, the ensuing fluorescence resonance energy transfer (FRET) to the attached PS produces cytotoxic reactive oxygen species that react with surrounding molecules (39).

In recent years, UCNPs usually containing rare-earth elements that exhibit upconversion properties have been developed for applications in biological labeling, sensing, and imaging (113). Idris and collaborators (81) carried out preclinical studies in which they tested the efficacy of mesoporous-silica–coated upconversion fluorescent nanoparticles, co-loaded with photosensitizers merocyanine and zinc (II) phthalocyanine, in melanoma cells, being the first research group to test UCNP-based targeted PDT *in vivo*. Tumor growth inhibition was observed in PDT treated mice, suggesting the procedure as a platform for future noninvasive deep-cancer therapy. The principle behind UCNPs allows the application of NIR light in PDT, permitting greater penetration depth in melanin-rich tissues, in comparison to VIS light (99, 111, 112), since light absorption by biomolecules is minimal in the NIR region. To illustrate this point, a study showed, in bovine muscle, that 37% of 500 to 600-nm light reached a depth of 4 mm, while this same amount of 800-nm light reached an 8 mm depth in this same tissue (114, 115). Concerning the production of ^1^O_2_, Wang *et al*. showed that the PS Ce6 alone, excitable by 660-nm light, did not produce ^1^O_2_ when irradiated in pork tissue at a depth of 8 mm; however, when associated to 980-nm light absorbing UCNPs, Ce6 produced decent levels of ^1^O_2_ at the same depth (116). These data confirm that NIR light penetrates deeper in biological tissues if compared with visible light. Other studies have also demonstrated UCNPs efficiency in the treatment of other types of cancer in *in vitro* and *in vivo* models, highlighting the promise of UCNPs for multifunctional cancer theranostics (39, 113, 117–119).

Some drawbacks for UCNP-based PDT, however, have yet to be properly addressed by future researchers, as discussed elsewhere (120). These drawbacks include: 1) low brightness; 2) excitation wavelength of 980 nm, commonly seen for most UCNPs, overlaps with the absorption of light by water, thus may bring tissue heating-related problems for UCNP-based PDT; and 3) biodegradability is quite low, requiring UCNPs to be smaller than 10 nm in diameter.

## Perspectives on the application of nanotechnology and PDT for the treatment of melanoma

As mentioned before, several types of nanomaterials have already been used along with PDT for the treatment of melanoma in *in vitro* and *in vivo* experimental models (Table 1), obtaining promising results. However, when it comes to completed clinical trials, these have focused either on the use of PDT for melanoma treatment or on the application of nanotechnology to improve treatment for this type of skin cancer. Most PDT clinical trials have focused on the treatment of choroidal melanoma and have used ranibizumab along with PDT in phase II study (NCT01251978); and indocyanine green (ICG) - based PDT in phase IV trials (NCT01253759). Another study combined the use of ranibizumab with ICG-based PDT in phase III trial (NCT00680225). One other trial involving the treatment of skin melanoma employed verteporfin for patients with stage III and IV melanoma (NCT00007969) (1).

**Fig. 3:**
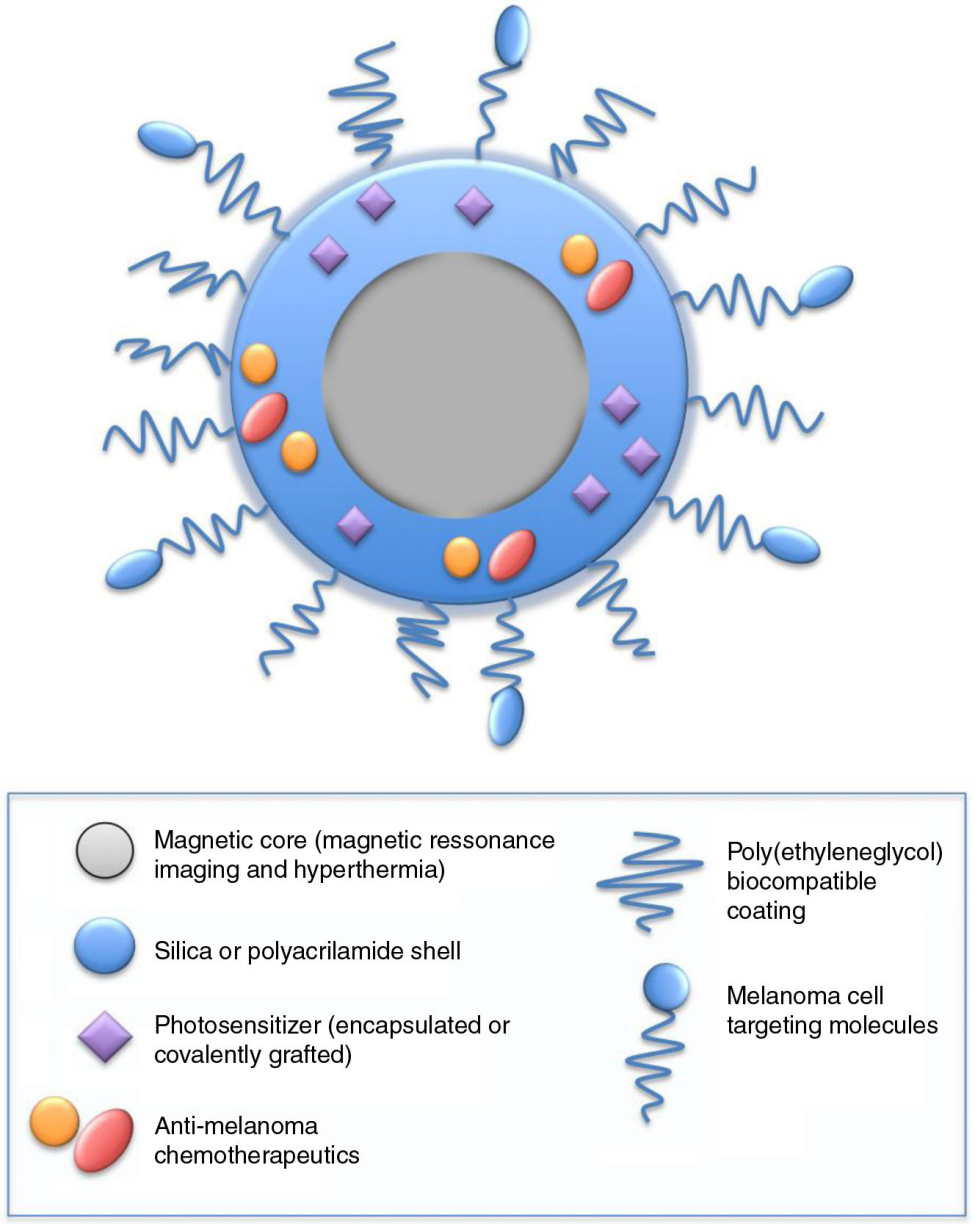
An ideal multifunctional nanosystem for the treatment of melanoma using photodynamic therapy (PDT), hyperthermia, site specific drug delivery, and targeting moieties. Photosensitizers and chemotherapeutics can be coupled or encapsulated to a silica or polyacrylamide shell for simultaneous PDT/chemotherapeutic treatment with site specific drug delivery. Nanoparticles can also be targeted to cells of interest or to tumor vasculature by surface-functionalization with targeting molecules. Contrast enhancers can also be incorporated as diagnostic agents.

Likewise, only a few trials have investigated nanomedicine for melanoma so far, but in view of the amount of preclinical work that is underway, it would be logical to assume that the number of clinical studies is set to dramatically increase in the next few years. Most of these nanomedicine clinical trials researching melanoma have explored the use of albumin-bound paclitaxel for unresectable skin and uveal tumors, using one chemotherapeutic (NCT00738361; NCT00081042) or a combination with other drugs (clinical trials NCT00626405; NCT00404235) (121). As mentioned before, it is known that nano-based therapeutics offer distinct advantages over conventional drug treatments in the sense that they can provide multifunctional combinations of targeting ability, diagnostic value, and therapeutic capacity, all in one unique particle (Fig. 3). Baldelli and colleagues (1) extensively explored this topic in their review article where they present several studies, mainly preclinical, focused on a variety of nanomaterials currently used to treat melanoma and strategies to improve nanoencapsulation and consequent delivery of common chemotherapeutics.

The next generation of nanoparticles will need to address the challenge to successfully reach not only the tumor cells themselves but also the different tissues and organs where metastases of late-stage tumors have spread. In the case of melanoma, metastases are present in a range of tissue types, and every tissue has its own unique challenges for nanoparticle penetration (1). Thereafter, it is possible that a promising solution to truly improve the efficacy of melanoma theranostics involves the combination of PDT and nanotechnology, perhaps through the use of multifunctional UCNPs and targeted PDT (39, 81, 113, 122). Moreover, the use of multimodal therapies leading to synergistic effect has become a promising approach to enhance anti-cancer therapy, reduce undesirable systemic toxicity and side effects (123). PDT along with magnetohyperthermia, photothermal therapy and electrochemotherapy using nanomaterials has also been studied with the perspective of achieving enhanced therapeutic cancer treatments (123–130). Magnetohyperthermia refers to a temperature rise caused by energy dissipation in the form of heat from magnetic nanoparticles that respond to an alternated frequency magnetic field. This temperature elevation initiates a series of subcellular events, rendering the cells to be susceptible to various forms of damage including apoptosis, leading to subsequent cell death (131, 132). Photothermal therapy is a laser-based technique that involves optical absorbing agents, such as gold nanostructures, carbon nanomaterials, various other inorganic and organic nanoparticles with strong NIR absorbance, to effectively convert photoenergy into heat to kill cancer cells under light irradiation (133). Electrochemotherapy combines electropulsation of tumor cells (by local application of electric pulses) and the administration of antineoplastic drugs such as cisplatin or bleomycin (either intravenous or intratumoral) (134). In relation to the application of these therapies for the treatment of melanoma, since magnetohyperthermia is not dependent on light, it may circumvent the problem of pigmentation. Photothermal therapy is dependent on light, but in this case NIR-light can be used, also avoiding extensive absorption by melanin. Electrochemotherapy enhances the penetration of chemotherapeutic drugs, and may be useful for increasing the penetration of PS as well. However, all of these therapeutic approaches still need to be better understood and standardized in *in vivo* and clinical studies. Future research should be oriented on this type of synergistic effects, along with the use of nanotherapies, in a way that melanoma treatment can in fact be more efficiently accomplished.

## Conclusion

When it comes to the treatment of melanoma, the outcomes of clinically available anticancer therapies are still very poor. Moreover, over the last decades, these therapies were not significantly improved. In face of this scenery, it is necessary to rethink the strategy and design of new tools for melanoma treatment taking into account the recent technological advances in the field of anticancer therapy. On that ground, PDT has been experimentally shown to strike cancer by different mechanisms, showing to be a good candidate for the treatment of different types of cancer. Although it has been clinically applied against a number of different cancer types with an encouraging therapeutic efficacy, its application against pigmented melanoma remains a challenge. Nanotechnology offers tools that enable improving conventional anticancer therapies. This is also the case when nanostructures are used for PDT against melanoma, as suggested by initial works on this subject. Therefore, PDT based on nanotech platforms may be a new approach for improving the treatment of melanoma.
